# Bone Marrow-Derived Cells as a Therapeutic Approach to Optic Nerve Diseases

**DOI:** 10.1155/2016/5078619

**Published:** 2015-11-16

**Authors:** Louise A. Mesentier-Louro, Camila Zaverucha-do-Valle, Paulo H. Rosado-de-Castro, Almir J. Silva-Junior, Pedro M. Pimentel-Coelho, Rosalia Mendez-Otero, Marcelo F. Santiago

**Affiliations:** ^1^Instituto de Biofísica Carlos Chagas Filho, Universidade Federal do Rio de Janeiro, 21941-902 Rio de Janeiro, RJ, Brazil; ^2^Instituto Nacional de Infectologia Evandro Chagas (INI), Fundação Oswaldo Cruz, 21040-900 Rio de Janeiro, RJ, Brazil; ^3^Instituto de Ciências Biomédicas, Universidade Federal do Rio de Janeiro, 21941-902 Rio de Janeiro, RJ, Brazil

## Abstract

Following optic nerve injury associated with acute or progressive diseases, retinal ganglion cells (RGCs) of adult mammals degenerate and undergo apoptosis. These diseases have limited therapeutic options, due to the low inherent capacity of RGCs to regenerate and due to the inhibitory milieu of the central nervous system. Among the numerous treatment approaches investigated to stimulate neuronal survival and axonal extension, cell transplantation emerges as a promising option. This review focuses on cell therapies with bone marrow mononuclear cells and bone marrow-derived mesenchymal stem cells, which have shown positive therapeutic effects in animal models of optic neuropathies. Different aspects of available preclinical studies are analyzed, including cell distribution, potential doses, routes of administration, and mechanisms of action. Finally, published and ongoing clinical trials are summarized.

## 1. Introduction

Optic neuropathy is an umbrella term encompassing a large number of disorders that cause optic nerve damage. The retrograde degeneration of axons of retinal ganglion cells (RGCs) within the optic nerve can ultimately lead to the death of RGCs, which have their cell bodies in the inner retina, culminating in irreversible visual loss [1]. Glaucoma, the leading cause of irreversible blindness worldwide, is a progressive neuropathy that results from mechanical axonal damage at the optic nerve head [2]. It has been estimated that 64.3 million people had glaucoma in 2013 and that this number will increase to 111.8 million in 2040 [3]. Although the etiology of glaucoma is still a matter of intense investigation, the following risk factors have been associated with the disease: elevated intraocular pressure, use of systemic or topical corticosteroids, advanced age, thinner central cornea, vascular dysregulation, myopia, larger optic disc, positive family history, and African or Afro-Caribbean origin. Currently, treatment of glaucoma is limited to medications and surgical or laser procedures that reduce intraocular pressure [4, 5].

In contrast to the progressive nature of glaucoma, acute optic neuropathies are characterized by the acute onset of visual loss and are usually caused by ischemia (ischemic optic neuropathies), traumatic brain injury (traumatic optic neuropathy), and infection or inflammation (optic neuritis). Other causes of optic nerve injury, with varied clinical presentations, are compression, toxic or nutritional causes, infiltration of neoplastic or inflammatory cells, and papilledema secondary to elevated intracranial pressure [1, 6, 7]. Optic neuropathy can also occur in hereditary neurodegenerative disorders related to primary mitochondrial dysfunction, as well as in two nonsyndromic mitochondrial hereditary optic neuropathies: Leber hereditary optic neuropathy and dominant optic atrophy. These two disorders have an estimated prevalence of 1 : 45,000 (in Europe) and 1 : 25,000 (in northern England), respectively [8–11]. Moreover, RGC death and optic nerve degeneration may occur in other highly prevalent neurological disorders, such as multiple sclerosis and Alzheimer's disease [12, 13].

After optic nerve injury, RGCs are unable to regenerate their axons and undergo apoptosis, mostly due to an intrinsic inability to regenerate but also due to the inhibitory environment of the central nervous system (CNS) [14, 15]. In order to stimulate neuronal survival and axonal outgrowth, many groups have been working on animal models of glaucoma and optic nerve injury. Strategies to improve regeneration include attempts to shift the inhibitory environment of the CNS to a permissive one and to stimulate the intrinsic regenerative programs of RGCs. For instance, it has been shown that RGCs are able to grow their axons on peripheral nerve grafts [16–18]. However, even though peripheral nerve grafting provides a permissive environment, it does not sustain RGC survival on a long-term basis after optic nerve transection [19].

More robust results have been obtained with the stimulation of RCG intrinsic regeneration program through, for example, the deletion of the phosphatase and tensin homolog (PTEN) or the suppressor of cytokine signaling 3 (SOCS3) [20–22]. After optic nerve injury, RGCs with deletion of both PTEN and SOCS3 have growing axons that form new synapses in the suprachiasmatic nucleus and reintegrate with the local circuitry [23]. Extensive regeneration has also been shown when adenoassociated virus (AAV) expressing short hairpin RNA against PTEN was coupled to AAV encoding ciliary neurotrophic factor (CNTF) and to a cyclic adenosine monophosphate (cAMP) analog [24]. The combination of PTEN deletion with the induction of inflammation through zymosan injection and elevation of intracellular cAMP has also led to long-distance regeneration and some evidence of functional recovery in this model [25, 26]. Moreover, using quantitative proteomics, Belin and coworkers revealed a network of signaling hubs following optic nerve injury and identified* c-myc* as a key regulator of the intrinsic regenerative mechanisms of RGCs [27].

Although these approaches are very promising, they are not easily translated to the clinic. The development of novel molecular tools for gene silencing has created an exciting new field of research, but there is still a long way to go before promising findings are translated into approved therapies, mainly due to safety issues that must be resolved [28, 29].

Cell therapy has emerged as a promising tool in regenerative medicine. Different research groups have used embryonic stem cells or induced pluripotent stem cells to generate RGCs that could replace the lost cells [30–32]. Although these newly generated cells express RGC markers, after injection into the vitreous chamber most of the transplanted cells remain close to the injection site, showing little capacity to integrate into the retina [30].

Another line of investigation has indicated that bone marrow-derived cells, such as mesenchymal stem cells (BM-MSCs) and mononuclear cells (BM-MNCs), could increase RGC survival and promote axonal regeneration after optic nerve injury in rodents [33–35]. In recent years, substantial experience has accumulated in the transplantation of BM-MSCs and BM-MNCs in patients with neurological disorders, indicating the safety and the feasibility of this approach [36].

This review summarizes and discusses the main findings of preclinical studies that have investigated the therapeutic action of bone marrow-derived cells in animal models of optic neuropathies. The use of noninvasive imaging methods to assess the distribution of the transplanted cells in the visual system and to investigate the efficacy of cell therapies is also discussed.

## 2. Bone Marrow Mononuclear Cells

The therapeutic potential of BM-MNCs has been extensively investigated in several disorders, including acute myocardial infarction and stroke [36, 37]. The mononuclear cell fraction is usually isolated from bone marrow aspirates by Ficoll-Paque density gradient centrifugation. Alternative isolation methods include the use of Percoll density gradient centrifugation or the immunomagnetic depletion of polymorphonuclear cells and erythrocytes [38]. BM-MNCs comprise a heterogeneous population of cells with diverse functions, including hematopoietic stem cells (HSCs), hematopoietic progenitors, endothelial progenitor cells (EPCs), mesenchymal stem cells (MSCs), monocytes, and lymphocytes. The bone marrow not only produces several types of immune cells but also attracts and retains different types of leukocytes. Zhao et al. [39] have elegantly reviewed the cellular composition of bone marrow, with an emphasis on the trafficking of immune cells.

Pang and colleagues [40] have shown that HSCs represented less than 0.20% of BM-MNCs in young individuals, although they were more frequent in the bone marrow of older individuals. HSCs are able to self-renew and differentiate into lineage-restricted progenitors, which subsequently give rise to the different blood cells [41]. CD34 is an antigen expressed by human HSCs and hematopoietic progenitor cells. Although CD34 can be expressed by other cell types, such as EPCs [42] and monocytes [43], the expression of this antigen has been used for the isolation of a human bone marrow cell population enriched in HSCs [44].

EPCs are circulating bone marrow-derived cells involved in endothelial repair and postnatal angiogenesis, due to their capacity to differentiate into mature endothelial cells and to secrete soluble factors, such as insulin-like growth factor 1 (IGF-1) and vascular endothelial growth factor (VEGF). They express HSCs and endothelial cell markers and can be cultured and expanded* in vitro* after isolation [42, 45]. Interestingly, there is increasing evidence that endothelial dysfunction associated with an altered number of circulating EPCs might play a role in the pathogenesis of neurological disorders, such as Alzheimer's [46] and Parkinson's [47] diseases.

In addition, BM-MNCs contain 0.001%–0.01% mesenchymal stem cells (MSCs) that can be expanded in culture [48]. In view of the importance of MSCs for regenerative medicine, these cells are described in detail below.

## 3. Mesenchymal Stem Cells

MSCs were first described by Friedenstein and coworkers [49], who observed fibroblast-like cells that adhered to plastic when bone marrow suspensions were plated. These cells were defined as colony-forming unit multipotent cells and were able to differentiate into adipocytes, chondrocytes, and osteoblasts [49].

Because they adhere to plastic, MSCs can be easily isolated by plating the mononuclear cell fraction or even the whole bone marrow suspension in tissue culture flasks. All contaminating nonadherent cells are removed after serial medium changes.

MSCs are characterized by the panel of positive and negative cell surface markers proposed by the International Society for Cellular Therapy in 2006 [50]. The MSC population is defined as >95% positive for CD105, CD73, and CD90 and >95% negative for CD45, CD34, CD14 or CD11b, CD79*α* or CD19, and HLA-DR. As previously mentioned, MSCs must be adherent to plastic and must be able to differentiate into chondrocytes, adipocytes, and osteocytes. Other surface markers are also expressed by MSCs, such as CD44, CD166, Stro-1, CD106, and CD146 [51].

MSCs occupy anatomically distinct locations within the bone marrow and are also found in endosteal, stromal, and perivascular niches [52–54]. Physiologically, MSCs support the HSC niche, protecting HSCs from apoptotic stimuli and preventing their differentiation [55]. MSCs can also be found in other tissues, including adipose tissue, dental pulp, umbilical cord, and placenta [56].

MSCs have the capacity to migrate to sites of injury following their intravascular administration. This process depends on molecules present on the surface of MSCs and endothelial cells, such as P-selectin and integrins [57]. After adhering to the endothelium, MSCs are capable of crossing it in a metalloprotease-dependent manner [58].

Interestingly, MSCs are considered to be not inherently immunogenic, as they express low levels of HLA class I antigens and do not express, or express in negligible levels, HLA class II antigens as well as their costimulatory molecules such as CD80, CD86, and CD40 [59]. This characteristic allows their allogeneic transplantation, with little or no risk of rejection. Furthermore, MSCs can release several immunomodulatory mediators and can attract immune cells through the release of chemokines [60].

The current view is that MSCs can exert neuroprotective and proregenerative effects, mainly by secreting multiple factors that act in a paracrine fashion [61, 62]. These beneficial effects can be observed in animal models of several neurological disorders, including Huntington's disease [63], stroke [64–66], and epilepsy [67].

## 4. Preclinical Studies

Several studies have described the therapeutic effects of bone marrow-derived cells in animal models of optic nerve disease. The main characteristics and the principal findings of these preclinical studies are summarized in Table 1. Since MSCs can be found in several tissues, a few studies using sources other than bone marrow are included.

Bone marrow cells have been tested in several animal models of glaucoma. Yu and coworkers [69] injected rat BM-MSCs intravitreally 2 weeks after the ligation of episcleral veins. They observed that more RGCs survived in the treated retinas and that these retinas expressed more basic fibroblast growth factor (bFGF) and CNTF [69].

Johnson et al. [71] and Harper et al. [70] used laser to cauterize the trabecular meshwork and injected BM-MSCs intravitreally. Both groups found a neuroprotective effect of BM-MSC transplantation. In addition, Harper has injected BM-MSCs that were engineered to secrete brain-derived neurotrophic factor (BDNF), which resulted in increased protection of RGCs. Importantly, these studies used different methods to estimate RGC survival and also used different doses and times of administration.

Emre and coworkers [68] increased the intraocular pressure in rats by injecting hyaluronic acid into the anterior chamber. One week after the induction, MSCs derived either from the bone marrow or from the adipose tissue were transplanted intravitreally. Retrogradely labeled RGCs were counted two and four weeks after cell transplantation, which showed that the number of RGCs was significantly increased at both time points in the treated animals compared to the untreated group. Furthermore, the authors found decreased levels of the cytokines interferon gamma (IFN-*γ*) and tumor necrosis factor alpha (TNF-*α*) in the treated retinas.

BM-MSCs also protected RGCs in a model of glaucoma that used laser pulses directed to the eye in order to block the aqueous outflow [73]. Interestingly, when BM-MSCs were transplanted into the anterior chamber after the induction of open angle glaucoma—differently from all the above-mentioned studies, in which BM-MSCs were injected into the vitreous body—the treatment improved the regeneration of the trabecular meshwork. This led to better control of the intraocular pressure and consequently to a decrease in RGC degeneration [74].

In a model of retinal ischemia and reperfusion, Li and coworkers [72] injected rat BM-MSCs into the vitreous body and found that the number of RGCs, compared to the untreated group, increased after 4 weeks. Treated retinas showed increased expression of bFGF, BDNF, and CTNF.

Several studies have used optic nerve crush or transection as a broad-spectrum model of diseases that affect the optic nerve. Our group, for instance, demonstrated that both rat BM-MNCs and BM-MSCs had therapeutic effects after optic nerve crush. Intravitreally injected BM-MNCs protected RGCs in the first 2 weeks (although the effect was lost at 4 weeks) and increased axonal outgrowth for at least 4 weeks after optic nerve crush. In addition, animals treated with BM-MNCs showed an increased expression of the immediate early gene NGFI-A in the superior colliculus after light stimulation, indicating full-length axonal regeneration and synaptic connection with neurons of the superior colliculus [34]. This finding was supported by the retrograde labeling of RGCs 2 months after optic nerve crush. DiI was injected in the SC of BM-MNC-treated and untreated animals, but only treated animals showed DiI-positive, exuberant RGCs in the retina. This finding indicated the complete regeneration of RGCs axons, allowing the transport of DiI from the axon terminal in the brain to the cell body in the retina [34].

On the other hand, in rats, intravitreal injection of BM-MSCs led to sustained neuroprotection of RGCs for at least 4 weeks after optic nerve crush, which was the longest time period analyzed. Axonal outgrowth was also increased in these animals at the two time points analyzed (2 and 4 weeks). The association of sustained RGC survival and axonal regeneration in BM-MSC-treated animals suggests that RGCs may find favorable conditions to regenerate over longer distances and reconnect to their targets, as observed after BM-MNC treatment. This possibility is currently being investigated. Although BM-MNCs and BM-MSCs remained mostly in the vitreous body, treated animals showed increased expression of bFGF in their retinas. The antiapoptotic Tax1-binding protein 1 (Tax1BP1) and synaptotagmin IV gene expression were also upregulated in BM-MNCs treated retinas, while IL-1*β* protein levels were increased in BM-MSCs-treated retinas [34, 35, 75].

In another recent study, BM-MSCs were injected intravitreally after optic nerve crush and promoted an increase in axonal regeneration, in a dose-dependent manner [77]. An elegant study performed by Mead and coworkers [62] compared the effects of BM-MSCs with MSCs extracted from dental pulp (DP-MSCs), injected intravitreally after optic nerve crush. Although both cell populations were protective for RGCs and stimulated axonal regeneration, DP-MSCs were more efficient. The authors showed that DP-MSCs secreted larger amounts of nerve growth factor (NGF) and BDNF than BM-MSCs, indicating that trophic factor release determined the magnitude of the effect. Specific and unspecific blockage of Trk receptors A, B, or C significantly reduced the neuroprotective effect, suggesting a possible key role for neurotrophic factors that bind to this family of receptors, especially NGF, BDNF, and neurotrophin-3, in the effects of MSCs therapies [62].

MSCs derived from human umbilical cord blood (hUCB-MSCs) were also tested as an alternative source. Zhao and coworkers injected hUCB-MSCs 7 days after optic nerve crush and observed improved RGC survival up to 28 days after the injury. They also observed increased levels of BDNF and glial cell line-derived neurotrophic factor (GDNF) in the retina after transplantation [78]. Similarly, Jiang and coworkers [79] transplanted hUCB-MSCs shortly after optic nerve crush and reported an increased survival of RCGs in treated animals. They also showed that treated animals had smaller decreases in amplitude and smaller increases in peak latency of the flash visual evoked potentials waveform compared to untreated animals and demonstrated the upregulation of GRP78 and downregulation of CHOP mRNA levels, suggesting that hUCB-MSCs could play a role in reducing endoplasmic reticulum stress [79].

Chen and coworkers, on the other hand, reported a transient effect of grafted hUCB-MSCs. Twenty-one days after injury, both RGC survival and GAP-43 expression increased in the retina of treated animals, but these differences were no longer present after 28 days [80].

A different perspective was provided by Johnson and coworkers [61], with a coculture model using retina and BM-MSCs. When retinas are removed from the eye to be cultured* in vitro*, RGC axons are axotomized and these cells progressively die. Coculture with BM-MSCs increased RGC survival, and analysis of the secretome of BM-MSCs indicated the presence of several growth factors, especially those of the platelet-derived growth factor (PDGF) family. Blocking of the PDGF signaling pathway abolished the neuroprotection conferred by BM-MSCs in the coculture system. Moreover, the intravitreal injection of PDGF homodimers (PDGF-AA) or heterodimers (PDGF-AB) after experimental elevation of the intraocular pressure reduced the degeneration of RGCs axons in the optic nerve [61].

In addition to MSCs, the bone marrow includes other cell types with potential therapeutic effects on the visual system. Monocytes represent a population of circulating bone marrow-derived cells that play important roles in vascular and tissue homeostasis, as well as in the responses to pathogens, toxins, and other types of insults [81, 82].

Monocytes are recruited to the ganglion cell layer and the inner plexiform layer, where they differentiate into macrophages, in the first days following retinal intoxication with glutamate, a murine model of RGC death. The intravenous injection of bone marrow-derived monocytes, 1 day after injury, promoted the survival of Brn3a^+^ RGCs 7 days after the injury. This neuroprotective effect was further confirmed by Fluoro-Gold labeling of surviving RGCs. In contrast, the transfer of IL-10-deficient monocytes had no effect on RGC survival, indicating that the main mechanism of action of the transplanted monocytes was related to the release of this anti-inflammatory cytokine. In addition, transferred monocytes increased the number of proliferating neural progenitors in the ciliary body, although there was no evidence of neurogenesis [83].

A recent study, however, found that only 0.5–1% of the microglia/macrophages in the damaged retina came from circulating monocytes, 7–14 days after optic nerve transection in mice [84]. These findings suggest that the conditions for monocyte recruitment, such as alterations in the blood-brain barrier and the production of certain chemokines, might not be present in all types of optic neuropathies.

One strategy to stimulate optic nerve regeneration is the induction of intravitreal inflammation by injuring the lens or by intraocular injection of zymosan, a yeast cell wall preparation [85]. The proregenerative effect of zymosan seems to be mediated by the recruitment of neutrophils and macrophages (probably of monocyte origin) into the vitreous. Both cell types secrete oncomodulin, a growth-promoting factor for RGCs [86, 87]. However, Huang et al. [88] observed that whereas zymosan injection was able to improve the survival of RGCs after optic nerve axotomy in F344 rats, the treatment was detrimental to RGCs in a model of acute intraocular pressure elevation in this rat strain. Further investigation is therefore needed to elucidate the functional plasticity of monocytes [89] and the multifaceted role played by monocyte-derived macrophages in the visual system, under pathological conditions.

### 4.1. Doses and Routes of Administration

Concerning the route of administration, most groups have injected the cells intravitreally. In one study, the systemic administration of BM-MSCs had no effect after experimental glaucoma induction, while the intravitreal injection protected RGCs and delayed axonal degeneration [71]. Interestingly, intravenously transplanted MSCs can be magnetically targeted to the retina. Yanai and coworkers labeled BM-MSCs with superparamagnetic iron oxide particles (SPIONs). The cells were injected into the tail vein and a gold-plated neodymium disc magnet was placed within the orbit to attract the cells to the eye [90]. This approach might represent an alternative to intravitreal or subretinal injections.

Cell doses were heterogeneous among the studies. Beneficial effects were observed with doses ranging from 30,000 to 500,000 BM-MSCs, while the BM-MNC dose used in our study was 5,000,000 cells (Table 1). Only a few studies have compared different doses of MSCs. In one study, the higher dose of MSCs was more efficient in improving the survival of RGCs, although the lower dose was also neuroprotective [73]. Similarly, another study found that a higher dose was more efficient in promoting optic nerve regeneration compared to a lower dose, although both doses were beneficial [77].

These observations suggest that the effects of MSCs are dose-dependent, but, to our knowledge, no studies have established the maximum tolerated dose for intraocular transplantation. There is a limit to the volume that can be injected into the vitreous body without causing damage to the eye, and therefore the number of injected MSCs cannot be too high.

Since we have observed that the neuroprotection conferred by BM-MNCs decays over time and that most of the transplanted cells are cleared from the vitreous body within 2 weeks, we investigated whether a second administration would change this outcome. We found that, even with a second dose, neuroprotection is lost over time. Axonal regeneration, however, was improved after the second injection, suggesting that BM-MNCs may provide neuroprotection and stimulate axonal outgrowth through different pathways [76].

### 4.2. Distribution and Persistence of the Transplanted Cells

In regenerative medicine, it is essential to determine where and how long the cells remain in the host tissue after transplantation and whether this phenomenon contributes to therapeutic effects.

Several studies have tracked bone marrow cells after injection in animal models of visual diseases. Yu and coworkers injected green fluorescent protein (GFP) expressing BM-MSCs into the vitreous body, after inducing glaucoma by ligation of episcleral veins in adult rats. Two weeks after the injection, they found GFP-positive cells along the inner limiting membrane, with few of them integrated into the ganglion cell layer. Transplanted cells were found in the host tissue for up to 8 weeks.

Interestingly, Na and coworkers found that when BM-MSCs were transplanted into normal eyes, they remained in the vitreous cavity. However, in ischemia/reperfusion injured retinas, BM-MSCs were found along the inner limiting membrane, and few of them were integrated into the ganglion cell layer, 4 weeks after injection [72].

In glaucomatous eyes, induced by photocoagulation of the trabecular meshwork, the majority of the BM-MSCs remained in the vitreous body, occasionally attached to the posterior lens capsule, and a small number of them reached the nerve fiber layer and the ganglion cell layer [71]. Harper and coworkers [70] described a similar profile of BM-MSC grafting in glaucomatous eyes, while Emre and coworkers [68] reported that BM-MSCs or adipose tissue-derived MSCs integrated into the ganglion cell layer and inner nuclear layer when the intraocular pressure was raised by injecting hyaluronic acid.

Johnson and coworkers [91] also developed* in vitro* coculture system to investigate the engraftment of BM-MSCs in the retina. Similar to* in vivo* experiments, in this coculture system the BM-MSC grafts remained adjacent to the inner limiting membrane and did not integrate into the neural retina. In this study, the administration of a gliotoxic glutamate analogue enhanced BM-MSC grafting into the inner retinal layers, suggesting that glial cells are responsible for the poor integration of transplanted cells into the retina [91].

The use of histochemistry for cell tracking has many limitations. For example, misleading results can be obtained if the dye used to label transplanted cells is able to diffuse to retinal cells, as observed after labeling BM-MSCs with 4′,6-diamidino-2-phenylindole (DAPI) [92, 93].

The use of noninvasive* in vivo* imaging methods may improve the understanding of numerous questions in the field of cell therapies for optic nerve diseases, including aspects such as the evaluation of cell distribution. Firstly, the retina may be examined through the clear cornea and lens by ophthalmoscopy [94]. Scanning laser ophthalmoscopy (SLO) employs laser light at a certain wavelength to scan the retina [95]. Different SLO equipment allows confocal SLO (cSLO), which has millimetric resolution and may be used to visualize graft size [94]. However, other methods are required to distinguish transplanted cells in the eye and any possible migration outside the eye. One of the possible methods is to transfect the cells with reporter genes for fluorescence imaging (FLI). This approach indicates the presence of cells that are metabolically active expressing the reporter gene [96]. Fischer and colleagues transfected human BM-MSCs with a plasmid expression vector encoding enhanced GFP (eGFP) and incorporated the cells into miniaturized alginate spheres (MicroBeads) [97]. Then, the MicroBeads were subretinally implanted and tracked using cSLO [97]. The authors reported that eGFP-expressing cells encapsulated in MicroBeads continued to be viable for up to 4 months [97]. FLI may also be applied to macroscopically investigate if migration outside the eye takes place after intravitreal cell injection [98]. However, although the use of reporter genes is important for experimental investigations of cell migration, further studies are necessary to establish the safety of using viruses for transfection [99, 100].

Another method to macroscopically evaluate cell migration is to directly label cells with a radionuclide for nuclear medicine imaging or an exogenous contrast agent for MRI. Radionuclide cell labeling has been used for decades to diagnose infections through evaluation of the migration of labeled leukocytes by Single Photon Emission Computed Tomography (SPECT) [101]. More recently, SPECT has been applied for tracking cell therapies in different preclinical and clinical studies, with radionuclides such as Technetium-99m (^99m^Tc). PET may also be used for cell tracking, notably with fluorine-18 radiopharmaceuticals [96]. SPECT and PET may be acquired with conventional computed tomography (CT) and in combination as hybrid SPECT/CT or PET/CT. Such hybrid images permit simultaneous evaluation of functional and morphological information [102, 103]. Nevertheless, radiopharmaceutical cell labeling has restrictions, such as its relatively narrow time window for imaging. ^99m^Tc, the most frequently used radionuclide, has a 6-hour half-life and permits imaging for approximately 24 hours [101].

Contrast agents for MRI may surmount many of the present limitations of radiopharmaceuticals. SPIONs were originally created as intravenous contrasts for hepatic imaging and more recently have been used by different groups for cell labeling. One advantage of SPIONs is that the iron in the particles can be tracked by MRI* in vivo* for several days or weeks and also* ex vivo* by histochemistry (e.g., prussian blue stain). In addition, SPIONs can be detected by immunofluorescence against a polysaccharide (dextran) used for particle coating. Nonetheless, SPIONs have limitations, such as the possibility of dilution after cell proliferation, as well as sequestration of the iron from dying cells by macrophages or microglia [104].

To combine the advantages of SPECT/CT and MRI cell tracking, our group has investigated the distribution of BM-MNCs, using cell labeling with SPIONs (FeraTrack, Miltenyi Biotec, Germany) and ^99m^Tc in a model of optic nerve crush [76]. Immediately after the lesion, 5 × 10^6^ labeled cells or 5 *μ*L of saline was injected intravitreously. SPECT/CT was performed in the animals that received ^99m^Tc-labeled BM-MNCs, 1 hour after the cell transplant, and MRIs were acquired in animals that received SPION-labeled BM-MNCs up to 14 days after the cell therapy. Short-term tracking with SPECT/CT indicated that ^99m^Tc-labeled BM-MNCs were restricted to the eye and did not enter the bloodstream after injection [76]. However, long-term tracking with MRI indicated that the signal from SPION-labeled BM-MNCs had decreased 5 days after intravitreal injection and was almost absent at 14 days [76]. This was confirmed after labeling BM-MNCs with a fluorescent dye, which indicated that the number of cells was already reduced at 3 days after the injection [76] and that the cells were absent after 14 days [75]. On the other hand, BM-MSCs labeled with SPIONs were found* in vivo* inside the eye for up to 18 weeks and were also observed* ex vivo*, found predominantly in the vitreous body [35].

Taken together, these results suggest that while BM-MNCs injected into the vitreous body are cleared from this region within the first 2 weeks [34, 75], BM-MSCs can remain at the site for several weeks [35]. Despite their different temporal distributions, BM-MNCs and BM-MSCs were found mostly in the vitreous body, with poor integration into the inner retinal layers [34, 35]. Interestingly, there was an important difference in the duration of therapeutic effects between BM-MNC and BM-MSC therapy: while the neuronal protection observed in BM-MNC-treated animals was lost from 2 to 4 weeks after injury [76], BM-MSC-treated animals showed increased neuronal survival for at least 4 weeks after injury and cell transplantation [35]. These findings suggest a possible correlation between the persistence of transplanted cells at the injection site and their beneficial effects.

Several studies suggest that bone marrow-derived cell grafts act in a paracrine fashion and that neurotrophic factors play a role in the therapeutic effect. It is possible that the paracrine effect would be enhanced if transplanted cells could remain longer in the damaged tissue, given the progressive nature of several optic neuropathies. However, the length of time that the presence of grafted cells is necessary to sustain neuronal survival and/or to stimulate regeneration remains to be investigated. It is also important to determine whether once these goals are achieved, the permanence of transplanted cells would somehow impair visual function by, for example, eliciting a sustained inflammatory response in the eye.

While intravitreally transplanted MSCs may remain for several months inside the eye, different results were observed when BM-MSCs were injected into the anterior chamber in a model of glaucoma. They were cleared from the tissue within 96 hours, probably phagocytosed by microglial cells, since about 20% of the transplanted cells expressed the microglia/macrophage marker F4/80 on day 2. In spite of the short time they remained, MSCs were able to induce the regeneration of the damaged trabecular meshwork [74]. In our experience, intravitreally injected MSCs remained in the vitreous body for more than 4 months without expressing the microglia/macrophage marker Iba1, although Iba1-positive cells were found in their vicinity [35]. It remains to be determined whether these differences could be attributed to the site of injection or if MSCs are able to remain in the damaged area only as long as they are needed to repair the tissue. Evidence against the latter hypothesis was provided by Haddad-Mashadrizeh and collaborators, who showed that adipose-derived MSCs injected into the vitreous cavity of the intact eye remained in the ocular tissue for up to six months, suggesting that these cells are not cleared from the tissue, even in the absence of an injury [105].

### 4.3. Evaluation of Structural Effects Using Noninvasive Methods

In addition to analyzing cell distribution, noninvasive imaging methods also allow the assessment of structural parameters that can be used in the investigation of the safety and efficacy of cell therapies. Fischer and coworkers used cSLO to study ocular integrity and evaluate possible modifications in the anatomy of the cornea, lens, vitreous, and retina after a subretinal injection of MicroBeads containing eGFP-expressing BM-MSCs [97]. The authors reported that when several MicroBeads per eye were implanted into the subretinal space, significant retinal detachment and disruption of retinal integrity were seen in cSLO [97]. Optical coherence tomography (OCT) is another powerful imaging method that employs light to acquire three-dimensional images and allows retinal and optic nerve evaluation at a micrometer resolution in preclinical and clinical settings [95]. Fischer and colleagues were able to detect single MicroBeads and demonstrate their structural integrity [97]. On the other hand, Mead and coworkers used OCT as a tool to study the efficacy of intravitreally injecting BM-MSCs or DP-MSCs in adult rat RGCs after optic nerve crush [106]. One, two, and three weeks after the lesion, OCT was carried out to quantify the retinal nerve fiber layer width as a measure of axonal atrophy [106]. The authors reported that DP-MSCs conserved retinal nerve fiber layer for up to 14 days after optic nerve crush, while BM-MSCs had no effect on this parameter [106].

MRI is an important tool for the evaluation of the CNS and may be performed with or without the use of contrast agents such as gadolinium-diethylenetriamine pentaacetic acid (Gd-DTPA) and manganese (Mn^2+^) [107]. However, Gd-DTPA accumulates in regions of disrupted blood-brain barrier and frequently does not reveal noninflammatory diseases of single-fiber projections [108]. Mn^+2^-enhanced MRI (MEMRI) was developed to allow fiber tract imaging. Mn^2+^ is a calcium analog that is incorporated into neurons and is actively transported along preserved microtubules, whereas lesions stop its propagation [108]. Preclinical MEMRI of different CNS structures is possible after subcutaneous [109], intraperitoneal [110], intravenous [111], aerosolized [112], intravitreal [113], and topical [114] administration.

Haenold and collaborators [108] acquired high-resolution MEMRI by intravitreal injection of MnCl_2_ immediately after optic nerve crush in mice. They reported that axonal transport was reduced and interrupted proximal and distal to the nerve crush, respectively. Based on previous observations that unstimulated lesioned RGCs undergo limited fiber regeneration spontaneously [115], the authors carried out MEMRI and confirmed limited long-term regeneration one year after optic nerve crush [108]. To our knowledge, no studies with MEMRI have yet been carried out to evaluate the effects of bone marrow-derived cell therapies in optic nerve diseases. However, Mansergh et al. used MEMRI to evaluate the therapeutic potential of the intravitreal transplantation of retinal progenitor cells in a mouse model of Leber hereditary optic neuropathy [116]. The authors observed significant differences in signal intensity of MEMRI in animals treated with retinal progenitor cells at 1 and 3 months, indicating improvement after cell therapy [116].

However, the use of MnCl_2_ has limitations, because overexposure to Mn^2+^ causes neurological toxicity in humans and animals [113, 117]. Thuen et al. reported that rat RGCs directly exposed to intravitreally injected MnCl_2_ die at dosages higher than 300 nmol infused Mn^2+^ [113].

Diffusion tensor imaging (DTI) is another valuable MRI technique that maps the random motion of water molecules and reflects CNS microstructural integrity and pathology [118]. Zhang et al. evaluated DTI in mice and reported that it detected axonal injury as early as 6 hours after optic nerve crush [118]. Thuen and coworkers investigated the combined use of MEMRI and DTI to detect axonal injury in rats and to evaluate the regenerative potential of intravitreally transplanting a peripheral nerve graft in the optic nerve [119].

### 4.4. Evaluation of Functional Effects Using Noninvasive Methods

Different parameters may be used to evaluate the functional effects of cell therapies in animal models of optic nerve injury, including optokinetic response, pupil light reflex (PLR), electroretinography (ERG), and visual evoked potentials (VEPs).

Optokinetic response may be analyzed to quantify the capacity of distinguishing spatial frequency and contrast sensitivity in animal models of glaucoma [120]. Mansergh and collaborators used a video camera to quantify the animals' slow tracking movements in response to moving stripes in a chamber and beneficial effects were seen at 1 and 3 months after cell therapy with retinal progenitors in a mouse model of Leber hereditary optic neuropathy [116].

PLR may be analyzed by computerized pupillometry [70]. Harper and coworkers used PLR to investigate the therapeutic effect of intravitreal therapy with BM-MSCs engineered to express BDNF and GFP (BDNF-BM-MSCs) or just GFP (GFP-BM-MSCs) in a rat model of chronic ocular hypertension [70]. PLR evaluation indicated increased therapeutic potential for functional improvement in eyes treated with BDNF-BM-MSCs in comparison to GFP-BM-MSCs at 42 days after the lesion. They also investigated the use of ERG, which measures the electrical response of the retina to visual stimuli with corneal electrodes [70, 121]. The analysis indicated that retinal electrical activity was preserved at 20 and 40 days, in rats that received BDNF-BM-MSCs [70].

VEP analyzes occipital lobe brain wave potential after visual stimuli [121]. The VEP is carried out using electrodes positioned over the occipital area of both hemispheres, one eye at a time [121]. The elongation of P100 latency is a frequent abnormality observed in optic nerve dysfunction [121]. Zhang and colleagues evaluated the VEP in rats following intravitreal injection of human umbilical cord blood-derived cells or BDNF 7 days after optic nerve crush [122]. The authors reported that VEP detection scores showed greater peak voltages and shorter peak latencies in the treated groups in comparison to the control group, indicating functional improvement after cell therapy [122].

### 4.5. Mechanisms of Action of Cell Therapies

Although the initial studies of cell therapy suggested that bone marrow cells could differentiate into neuronal cells [123–126], it was later shown that these findings could be attributed to cell fusion, rapid disruption of the actin cytoskeleton, and/or cellular toxicity [127–129].

In most of the preclinical studies discussed in the previous sections, neuroprotection was attributed to a paracrine effect, and no evidence of neural transdifferentiation of MSCs has been shown. In this respect, the therapeutic effect of transplanted cells could be related to the capacity of MSCs to alter the microenvironment of the injured tissue, through the release of trophic factors and inflammatory mediators [130]. This paracrine effect may occur without the need for the integration of MSCs into the neural retina.

BM-MSCs can secrete a variety of trophic factors [131] and our group showed that rat bone marrow cells express the mRNAs for BDNF, bFGF, CNTF, VEGF, and transforming growth factor alpha (TGF-*α*) even without stimulation [34]. Moreover, the expression of CNTF, GDNF, BDNF, bFGF, and hepatocyte growth factor alpha chain (HGF*α*) was observed in GFP-positive BM-MSCs engrafted in glaucomatous retinas [69], suggesting that the paracrine effect may contribute to the cell therapy effect.

In order to increase this paracrine activity, a few studies have engineered the cells before transplantation. Intravitreally injected rat or human BM-MSCs that were stimulated to secrete neurotrophic factors were found in clusters between the lens and the retina and remained in the eye for at least 3 weeks after optic nerve transection. In this study, human BM-MSCs were more neuroprotective than rat BM-MSCs, which is correlated with the higher secretion of BDNF and GDNF by human cells, although it is possible that a beneficial inflammatory reaction could have been elicited by the xenotransplant [33].

In addition to the release of neurotrophic factors that may act directly on damaged neurons, the interaction between transplanted cells and retinal glial cells cannot be ignored. Indeed, we have shown that BM-MNC transplantation was associated with a reduction in the expression of glial fibrillary acidic protein (GFAP) in radial glial processes throughout the retinal layers, which is a marker of Müller cell reactivity in response to injury [34]. On the other hand, a recent study used BM-MSCs and suggested that the transplantation of these cells increased glial reactivity in the retina [132]. The implication of cell therapies for retinal gliosis and the contribution of these changes to the observed therapeutic effects need further elucidation.

A large number of studies have demonstrated that BM-MSCs exert beneficial immunomodulatory effects by interacting with cells of the innate and adaptive immune system [130, 133]. Furthermore, BM-MSCs constitutively express several innate immune sensors, such as toll-like receptors (TLRs). These receptors recognize many molecules expressed/released by pathogens or released upon tissue injury, being involved in the initiation and regulation of immune responses [134]. For instance, TLR4-primed MSCs (also called MSC1) exhibit a proinflammatory profile, while TLR3-primed MSCs (MSC2) adopt an anti-inflammatory phenotype [135].

Several studies have suggested that the crosstalk between MSC and the injury microenvironment leads to the secretion of soluble factors by these cells [136]. For example, upon stimulation by proinflammatory cytokines such as TNF-*α* and IFN-*γ*, MSCs secrete immunosuppressive mediators, including prostaglandin E2 (PGE2), indoleamine 2,3-dioxygenase (IDO), transforming growth factor beta-1 (TGF-*β*1), and IL-10 [130]. The interaction with immune cells also takes place in the CNS. For example, MSCs cocultured with lipopolysaccharide-activated microglia reduced the expression of TNF-*α*, inducible nitric oxide synthase (iNOS), and oxidative-stress related proteins by microglia and augmented the expression of IL-1*β*, CX3CR1, NURR1, and EP2. Interestingly, the authors showed that the functional changes in microglia were induced by the release of the chemokine CX3CL1 by MSCs, switching microglia from a neurotoxic to a neuroprotective phenotype [137].

Besides the secretion of soluble factors, it has recently been suggested that the therapeutic effect of BM-MSCs may be partially related to the release of extracellular vesicles. These vesicles, named “exosomes” or “microvesicles” according to their size, contain proteins, mRNA, and microRNA that can be transported from one cell to another [136]. Proteins present in BM-MSC extracellular vesicles include signaling molecules such as mitogen-activated protein kinase 1 (MAPK1), cell adhesion mediators such as fibronectin, and surface receptors such as PDGF receptor. Extracellular vesicles derived from MSCs also express regulatory molecules such as membrane-bound TFG-*β*, galectin-1, and programmed death ligand-1 [138].

Considering the biological function of proteins and microRNAs transported by extracellular vesicles, it would be interesting to evaluate the therapeutic potential of BM-MSC extracellular vesicles in animal models of optic neuropathies. In a model of stroke, for example, Xin and coworkers [139] showed that the secretion of microRNA 133b, mediated by extracellular vesicles from BM-MSCs, contributed to the beneficial effects of cell therapy. In this model, BM-MSC exosomes were transferred to neurons and astrocytes [139].

## 5. Published Clinical Studies of Bone Marrow Cell Therapies for Optic Nerve Lesions

Jonas et al. published a case report of autologous BM-MNC therapy for a 43-year-old patient with advanced retinal and optic nerve atrophy due to diabetic retinopathy [140]. The authors performed an intravitreal injection of 0.5 mL containing 1.8 × 10^8^ BM-MNCs and concluded that the procedure was feasible and safe [140].

Connick and coworkers [141] carried out an open-label phase 2a study in patients with secondary progressive multiple sclerosis affecting the visual pathways. A mean dose of 1.6 × 10^6^ autologous BM-MSCs was injected intravenously in 10 patients with clinical and electrophysiological confirmation of optic nerve injury. The authors reported that there were no serious adverse events and described an increase in visual acuity, visual evoked response latency, and optic nerve area.

## 6. Ongoing Clinical Studies of Bone Marrow Cell Therapies for Optic Nerve Lesions

At least three studies registered in clinicaltrials.gov have been designed to carry out bone marrow cell therapy for optic neuropathies. Weiss and collaborators, from a private clinic in Florida, USA, started an open-label study in August 2013 with an estimated completion date in August 2017, where 300 patients are expected to receive autologous BM-MSCs (NCT01920867). Retrobulbar, subtenon, intravenous, intravitreal, and intraocular injections will be administered to patients with retinal disease, macular degeneration, hereditary retinal dystrophy, optic nerve disease, and glaucoma, respectively. The primary and secondary outcome measurements will be visual acuity and visual fields, respectively, and patients will be followed up for 12 months.

De Paula and coworkers, from the University of São Paulo, Brazil, have designed an open-label, single-group phase 1 study to perform intravitreal autologous transplantation of 10^6^ BM-MSCs in patients with retinal degeneration or primary open-angle glaucoma (NCT02330978). The reported that the start date was January 2014 and a total of 10 patients are programmed to be included, with an estimated study completion date of December 2016. Patients will be followed up for 6 months. Primary outcome measurements will be the type and severity of adverse effects. The secondary outcome measurements will be changes in visual acuity, visual field, OCT parameters, and RGC function as assessed by ERG.

Jamadar and colleagues, from Chaitanya Hospital in Pune, India, started in September 2014 an open-label, phase 1/phase 2 study (NCT01834079). A total of 24 patients with optic nerve atrophy will be enrolled to receive an intrathecal injection of 10^8^ autologous BM-MNCs per dose in three applications at intervals of 7 days. The estimated study completion date is July 2016 and patients will be followed up for 6 months. The primary outcome measurement will be reduction in degeneration of the optic nerve with improvement in vision. Secondary outcome measurements will be an increase in visual function and improvement in idiopathic intracranial hypertension.

Other trials of stem cell therapies for degenerative eye diseases, such as diabetic and ischemic retinopathy, which may secondarily affect the optic nerve, have been recently reviewed by Mead and collaborators [142].

## 7. Conclusion

Studies using different animal models of optic nerve injury, such as optic nerve compression or transection and elevation of the intraocular pressure, have shown that RGC degeneration can be reduced by intravitreal transplantation of BM-MSCs or BM-MNCs, which was the delivery method used most often. Doses varied among the studies, but few of them suggested that higher doses have increased therapeutic potential. Bone marrow-derived cell effects were mostly attributed to the release of soluble factors that can protect RGCs and/or modulate the inflammatory response in the retina. Interestingly, there is evidence of long-term persistence of BM-MSCs at the injection site, although the importance of this phenomenon remains to be elucidated. Such preclinical studies showing the neuroprotective and proregenerative effects of bone marrow-derived cells have encouraged the execution of phase 1 or phase 2 clinical trials for diseases that affect the optic nerve. Several trials are ongoing and a few have been concluded, indicating the feasibility and safety of intravitreal or intravenous administration of autologous bone marrow-derived cells. Most preclinical studies focused on morphological outcomes such as RGC survival and axonal outgrowth, but as these parameters were improved in treated animals, there is a growing need for visual functional analysis in future studies. Further investigations are also necessary to unravel the mechanisms of action of transplanted cells, in order to allow the development of safe and efficient therapies.

## Figures and Tables

**Table 1:** Summary of published preclinical studies.

Animals	Cell injection(s)	Effects, longest time analyzed after injury	Distribution, longest time analyzed after transplantation	Mechanisms	References
*Glaucoma models*: *rise of the intraocular pressure through several approaches, retinal ischemia/reperfusion*					

Adult female Wistar rats (MSCs were from male)	ivit rat BM-MSCs or rat AT-MSCs 1 w after injury **Dose**: 200,000 cells	**4 w**; RGCs retrogradely labeled, % to uninjured: BM-MSCs (83%); AT-MSCs (85%); untreated (65%)	4 w; cells were integrated into GCL and INL	Reduced TNF-*α* and interferon-*γ*; increased IL-1Ra and prostaglandin E2 receptor	[68]

Adult female Wistar rats	ivit rat BM-MSCs 2 w after injury **Dose**: 200,000 cells	**4 w**; RGCs retrogradely labeled, % to uninjured: BM-MSC (88%); untreated (80%)	8 w; ILM, NFL, GCL	Increased expression of bFGF and CNTF	[69]

Adult brown Norway rats (sex not specified)	ivit BDNF-expressing rat BM-MSCs; rat BM-MSCs 2 d after injury **Dose**: 200,000 cells	**6 w**; BDNF-BM-MSCs treated animals had better pupil light reflex, ERG function, and optic nerve integrity; RGCs (Brn3a-positive cells) % to uninjured: BDNF-MSCs (56%); MSCs (29%)	6 w; GCL, vitreous	Chronic low dose delivery of BDNF	[70]

Adult male Sprague-Dawley and Lewis rats	ivit and IV rat live or dead BM-MSCs 1 w before the injury **Dose**: 30,000 cells	**4 w**; intravenous: no effect. Intravitreal: RGCs survival (optic nerve damage): Lewis: 92% (live BM-MSC); 74% (dead BM-MSCs) Sprague-Dawley: 89% (live BM-MSCs); 62% (dead BM-MSCs)	5 w: majority of cells in the vitreous, few in the ILM, NFL, and GCL	n/a	[71]

Adult male Sprague-Dawley rats	ivit rat BM-MSCs **Dose**: 180,000	**4 w**: RGCs (nuclei counting in the GCL): 1.3 fold increase to untreated	4 w: vitreous, ILM, GCL	Increased expression of bFGF, CNTF, and BDNF	[72]

Aged male Sprague-Dawley rats	ivit BM-MSCs 6 w after injury **Dose**: low (30,000 cells); high (100,000 cells)	**10 w**: RGCs retrogradely labeled, % to uninjured: 40% (untreated), 58% (low dose); 69% (high dose) Improved visual water box swimming test (better in high dose)	n/a	n/a	[73]

Adult brown Norway female rats	ant/cha murine BM-MSCs **Dose**: 100,000 cells	**4 w**: trabecular meshwork regeneration; decreased IOP; decreased RGCs degeneration (TUNEL^+^ cells)	24 h: cells migrated to the damaged area 96 h: cells cleared	Secretion of paracrine factors; recruitment of ocular progenitor cells	[74]

*Optic nerve crush or transection*					

Adult male and female Lister hooded rats	ivit rat BM-MNCs immediately after injury **Dose**: 5,000,000 cells	**2 w**: RGCs retrogradely labeled, % to uninjured: BM-MNCs (40%); vehicle (24%); higher optic nerve regeneration; **8 w**: RGCs regeneration and reconnection to the superior colliculus	2 w: very few cells GCL, INL, optic nerve	Müller glia modulation; bFGF, Tax1BP1, and SytIV increased expression	[34, 75, 76]

Adult male Sprague-Dawley rats	ivit rat BM-MSCs **Dose**: low: 30,000; high: 100,000	**2 w**: higher optic nerve regeneration. High dose had more regeneration than low dose of BM-MSCs	n/a	n/a	[77]

Adult Wistar rats (sex not specified)	ivit NTF-secreting BM-MSCs; human BM-MSCs; rat BM-MSCs 3 d before the injury **Dose**: 400,000	**8 d**: RGCs retrogradely labeled, % to uninjured: human NTF-BMSCs (69%); human BM-MSCs (66%); vehicle (46%); rat BM-MSCs had no neuroprotective effect	24 d: vitreous	Neurotrophic factors secretion (GDNF, BDNF); possible inflammatory reaction to xenotransplantation	[33]

Adult male and female Lister hooded rats	ivit rat BM-MSCs **Dose**: 500,000	**4 w**: RGCs (Tuj1^+^ and Brn3a^+^ cells; % to uninjured): 21% and 5% MSCs; 9% and 2% vehicle	18 w: vitreous	Increased expression of bFGF and IL-1*β*	[35]

Adult male Sprague-Dawley rats	ivit rat DP-MSCs or rat BM-MSCs **Dose**: 150,000 cells	**3 w**: RGCs (*β*-III tub^+^ cells/mm): DP-MSCs (28), BM-MSCs (16), untreated (6.9); higher axon regeneration and RFNL thickness in both treated groups, best on DP-MSCs	3 w: DP-MSCs in the vitreous; BM-MSCs n/a	Neurotrophic factors release (NGF, BDNF, and NT-3); DP-MSCs release more NGF and BDNF than BM-MSCs; reduced scar tissue on the crush site	[62]

*In vitro*: *organotypical retinal explant culture*					

Adult Sprague-Dawley rats (sex not specified)	A droplet containing 1,000–5,000 BM-MSCs was placed on the RGCs surface	**1 w**: RGCs number increased ~2-fold (Islet-1; NeuN, *β*-III tub); increased NFL and IPL thickness	1 w: adjacent to GCL, not integrated into the retina	Secretion of growth factors, specially PDGF (also tested *in vivo* in a glaucoma model)	[61]

ant/cha: anterior chamber; AT-MSCs: adipose tissue-derived mesenchymal stem cells; BDNF: brain-derived neurotrophic factor; bFGF: basic fibroblast growth factor; BM-MNCs: bone marrow-derived mononuclear cells; BM-MSCs: bone marrow-derived mesenchymal stem cells; CNTF: ciliary neurotrophic factor; DP-MSCs: dental pulp-derived mesenchymal stem cells; GCL: ganglion cell layer; GDNF: glial cell line-derived neurotrophic factor; IL-1Ra: interleukin-1 receptor antagonist; ILM: inner limiting membrane; INL: inner nuclear layer; IOP: intraocular pressure; IPL: inner plexiform layer; IV: intravenous; ivit: intravitreal; NFL: nerve fiber layer; NTF: neurotrophic factors; PDGF: platelet-derived growth factor; RGCs: retinal ganglion cells; sub: subretinal; SytIV: synaptotagmin IV; Tax1BP1: Tax1-binding protein 1; TNF-*α*: tumor necrosis factor alpha.
